# Changes in biomarkers of redox status in serum and saliva of dogs with hypothyroidism

**DOI:** 10.1186/s12917-023-03586-4

**Published:** 2023-02-03

**Authors:** Luis G. González Arostegui, Alberto Muñoz Prieto, Luis Pardo Marín, Gregorio García López, Asta Tvarijonaviciute, Jose Joaquín Cerón Madrigal, Camila Peres Rubio

**Affiliations:** 1 Interdisciplinary Laboratory of Clinical Analysis (Interlab-UMU), Veterinary School, Campus of Excellence Mare Nostrum, University of Murcia, 30100 Murcia, Espinardo Spain; 2 Clinic for Internal Diseases, Faculty of Veterinary Medicine, University of Zagreb, Heinzelova 55, 1000 Zagreb, Croatia; 3 Department of Animal and Food Science, School of Veterinary Science, Universitat Autònoma de Barcelona, 08193 Cerdanyola del Vallès, Barcelona, Spain

**Keywords:** Antioxidants, Biomarkers, Dogs, Hypothyroidism, Oxidants, Oxidative stress

## Abstract

**Background:**

Hypothyroidism is the most common endocrine disorder diagnosed in dogs, leading to deleterious effects on a dog’s life quality. This study aims to evaluate changes in the redox status in canine hypothyroidism. For this purpose, a comprehensive panel of antioxidants and oxidants biomarkers were measured in serum and saliva of 23 dogs with hypothyroidism, 21 dogs with non-thyroidal illness, and 16 healthy dogs. Among the antioxidants, cupric reducing antioxidant capacity (CUPRAC), ferric reducing ability of plasma (FRAP), Trolox equivalent antioxidant capacity (TEAC), thiol, paraoxonase type 1 (PON-1) and glutathione peroxidase (GPx) were determined in serum and CUPRAC, ferric reducing ability of saliva (FRAS) and TEAC in saliva. The oxidant biomarkers included were total oxidant status (TOS), peroxide-activity (POX-Act), reactive oxygen-derived compounds (d-ROMs), advanced oxidation protein products (AOPP), and thiobarbituric acid reactive substances (TBARS) in serum and AOPP and TBARS in saliva.

**Results:**

Results showed a significantly higher TEAC, PON-1, GPx, TOS, POX-Act, and d-ROMs, and a significantly lower AOPP in serum of dogs with hypothyroidism. Meanwhile, significantly lower FRAS and AOPP were observed in saliva of dogs with hypothyroidism. Once salivary concentrations were corrected based on their total protein concentrations, the only analyte showing significant changes was TBARS which was significantly higher in dogs with hypothyroidism.

**Conclusions:**

Our results show that dogs with hypothyroidism present alterations in the redox status in both serum and saliva. This study should be considered a preliminary study and further research addressing these changes should be made using larger populations.

**Supplementary Information:**

The online version contains supplementary material available at 10.1186/s12917-023-03586-4.

## Background

Hypothyroidism is described as a deficiency in the synthesis of thyroid hormones, namely, tetraiodothyronine (thyroxin, T4) and triiodothyronine (T3), due to an impairment of the thyroid gland, commonly associated in dogs with idiopathic follicular atrophy or destruction of the gland as a consequence of lymphocytic thyroiditis [1–3].

Thyroid hormones have an important association with oxidative status [4]. In physiological conditions, thyroid hormones have positive effects on metabolism, as they influence both catabolic and anabolic reactions. However, these reactions can lead to oxygen consumption, which is a key factor in the development and synthesis of reactive oxygen species (ROS). On the other hand, thyroid hormones also play an important role in the antioxidant defense system, as they act as part of this system being enzymatic and non-enzymatic free radical scavengers [3–10].

Markers of oxidative stress associated with hypothyroidism have been studied in humans, showing a decrease in antioxidants and an increase in lipid peroxidation [4]. Namely, a decrease in superoxide dismutase activity (SOD), catalase activity, and an overall increase in thiobarbituric acid reactive substances (TBARS) were found in red blood cell lysates of humans with hypothyroidism [11–13], and these changes were attenuated after treatment with levothyroxine [12].

In humans, serum has been traditionally used to evaluate changes in the oxidative status of patients with different thyroidal diseases [14–17]. However, saliva is an easy-to-obtain, stress-free sample that has been used for the study of different biomarkers in other diseases and species [18–22]. To the author’s knowledge, the oxidative status has not been evaluated in serum and saliva of dogs with hypothyroidism previously.

We hypothesized that biomarkers of the redox status could change in serum and saliva of dogs with hypothyroidism. Thus, the objective of this study was to evaluate biomarkers of the redox status in serum and saliva from dogs with hypothyroidism and compare them with dogs with non-thyroidal illnesses and healthy controls. A comprehensive panel of redox biomarkers was measured, including antioxidants as, the cupric ion reducing antioxidant capacity (CUPRAC), ferric reducing ability of plasma (FRAP), Trolox equivalent antioxidant capacity (TEAC), thiol, paraoxonase type-1 (PON-1) and glutathione peroxidase activity (GPx), and oxidants as, the total oxidant status (TOS), peroxide-activity (POX-Act), reactive oxygen-derived compounds (d-ROMs), advanced oxidation protein products (AOPP) and TBARS in serum and CUPRAC, FRAS, TEAC, AOPP and TBARS in saliva.

## Results

### Patient characteristics

A complete description of the dogs included in the study is presented in Table 1. There were no statistically significant differences between groups related to age or sex.

**Table 1:** Detailed characteristics (breed, mean age, and body condition score (BCS) of the different groups included in the study

	**Hypothyroidism (*n* = 23)**	**Non-thyroid diseased (*n* = 21)**	**Control group (*n* = 16)**	***P-value***
Breed	- 7 Mongrel - 5 Beagle - 2 Golden Retriever - 1 Teckel - 1 Spanish Water dog - 1 Dalmatian - 1 Neapolitan Mastiff - 1 Pinscher - 1 Yorkshire Terrier - 1 Bodeguero Andaluz - 1 Swiss Shepherd - 1 Pomeranian	- 8 Mongrel - 5 Beagle - 2 French Bulldog - 2 Yorkshire Terrier - 1 Basset Hound - 1 Boxer - 1 Pomeranian - 1 Pug	- 11 Beagle - 2 Yorkshire Terrier - 1 Mongrel - 1 Greyhound - 1 Malinois	
Age, mean (range)	9.78 years (7 – 14)	8.14 years (2 – 12)	7.35 years (5 – 14.6)	> 0.05
BCS, mean (range)	4.08 (3 – 5)	3.81 (2 – 5)	3.31 (2 – 4)	0.013 (Hypothyroid vs control)
Sex (Female /male)	20 males 3 females	13 males 9 females	13 males 3 females	> 0.05

### Serum

#### Antioxidant status

**Fig. 1:**
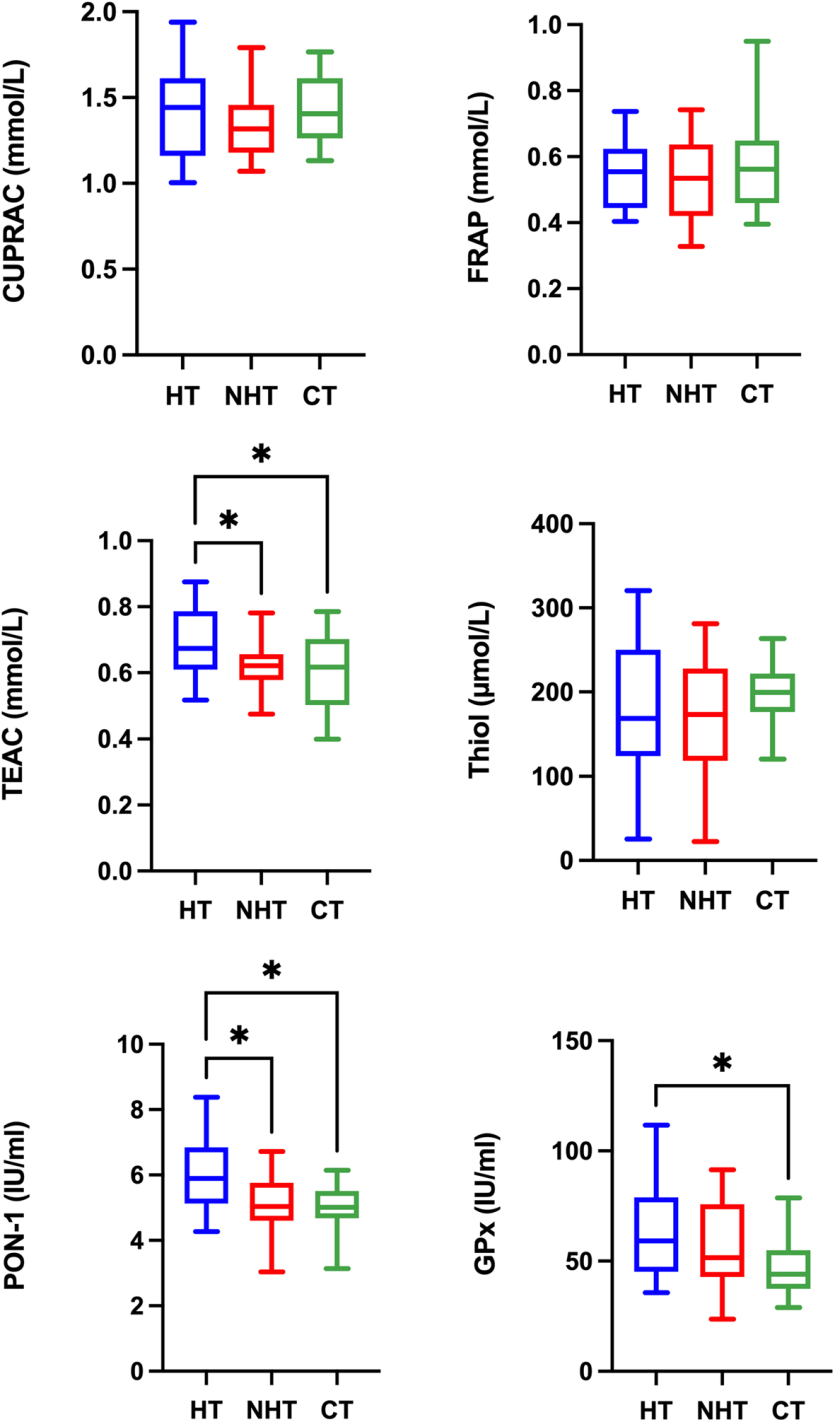
Results of antioxidant biomarkers in serum. Cupric reducing antioxidant capacity (CUPRAC); ferric reducing ability of plasma (FRAP); Trolox equivalent antioxidant capacity (TEAC); thiol, paraoxonase type-1 (PON-1), and glutathione peroxidase (GPx) in dogs with hypothyroidism (HT), non-thyroid diseased (NHT) dogs and controls (CT). Asterisks indicate significant differences between groups. *P﻿ ≤ 0.05

Results for the serum antioxidant biomarkers are shown in Fig. 1. TEAC and PON-1 showed significantly higher values in dogs with hypothyroidism compared to dogs with non-thyroid diseases (*P* = 0.0486 and *P* = 0.0069, respectively) and healthy dogs (*P* = 0.0420 and *P* = 0.0074, respectively). GPx activity showed significantly higher values in dogs with hypothyroidism compared to healthy dogs (*P* = 0.0344). No significant differences (*P* > 0.05) were found between groups in CUPRAC, FRAP, and thiol.

#### Oxidant status

**Fig. 2:**
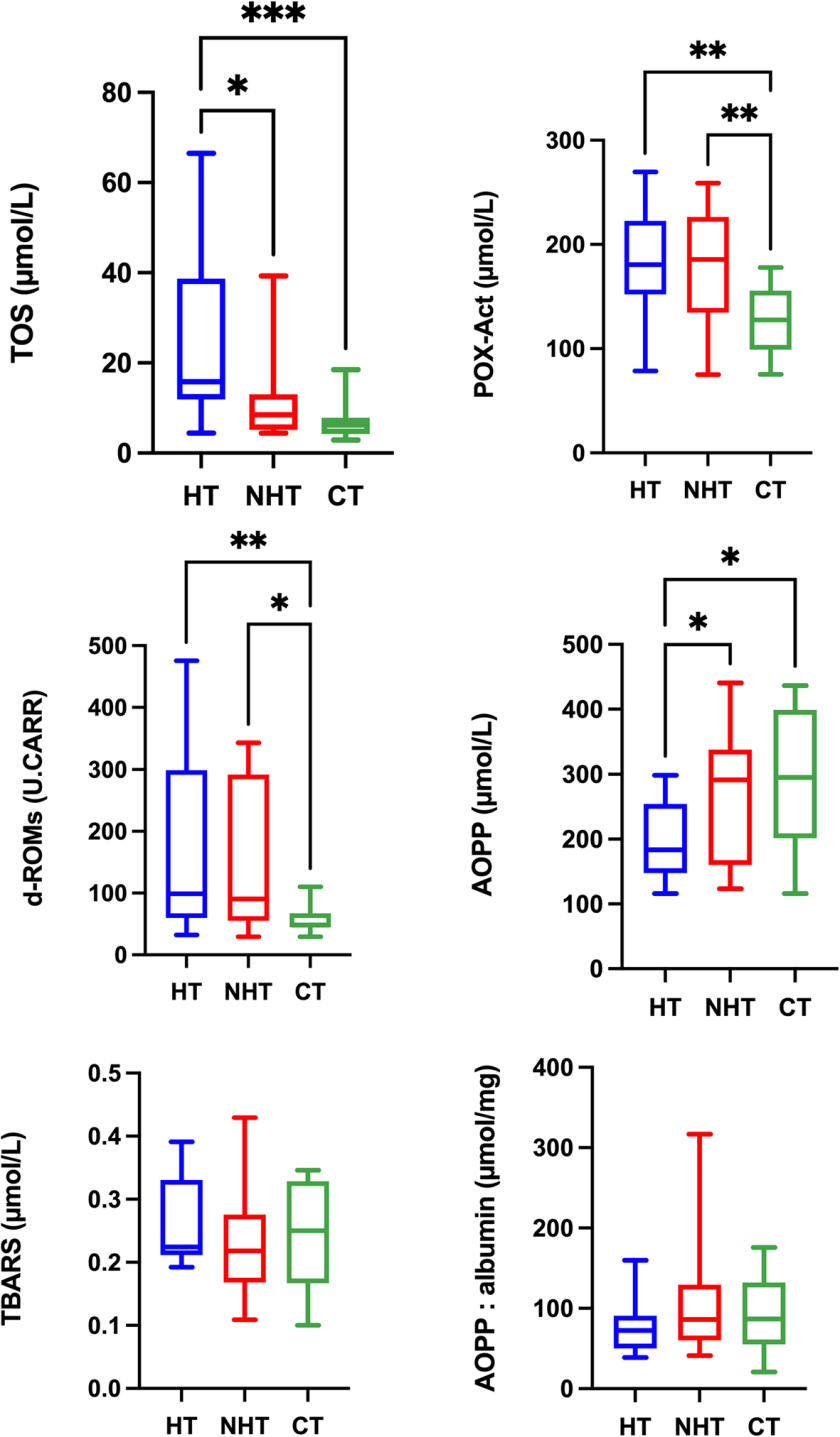
Results of oxidant biomarkers in serum. Total oxidant status (TOS); peroxide-activity (POX-Act); reactive oxygen-derived compounds (d-ROMs); advanced oxidation protein products (AOPP), thiobarbituric acid reactive substances (TBARS) and AOPP: albumin ratio in dogs with hypothyroidism (HT), non-thyroid diseased (NHT) dogs and controls (CT). Asterisks indicate significant differences between groups. *P﻿ ≤ 0.05, **P ﻿ ≤ 0.01, ***P﻿ ≤ 0.001

Results for the oxidant biomarkers for serum are shown in Fig. 2. TOS concentrations were significantly higher in dogs with hypothyroidism compared to dogs with non-thyroid diseases *P* = 0.0327) and healthy dogs (*P* = 0.0004). In addition, concentrations of POX-Act and d-ROMs were significantly higher in dogs with hypothyroidism (*P* = 0.0003) and *P* = 0.0066, respectively) and dogs with non-thyroid diseases (*P* = 0.0070) and *P* = 0.0280, respectively) in comparison to healthy dogs.

Concentrations of AOPP before albumin correction were significantly lower in dogs with hypothyroidism in comparison to dogs with non-thyroid diseases (*P* = 0.031) and healthy dogs (*P* = 0.0143). However, no significant differences were found (*P* > 0.05) between groups in AOPP and TBARS when results were corrected by albumin concentrations.

### Saliva

#### Antioxidant status

**Fig. 3:**
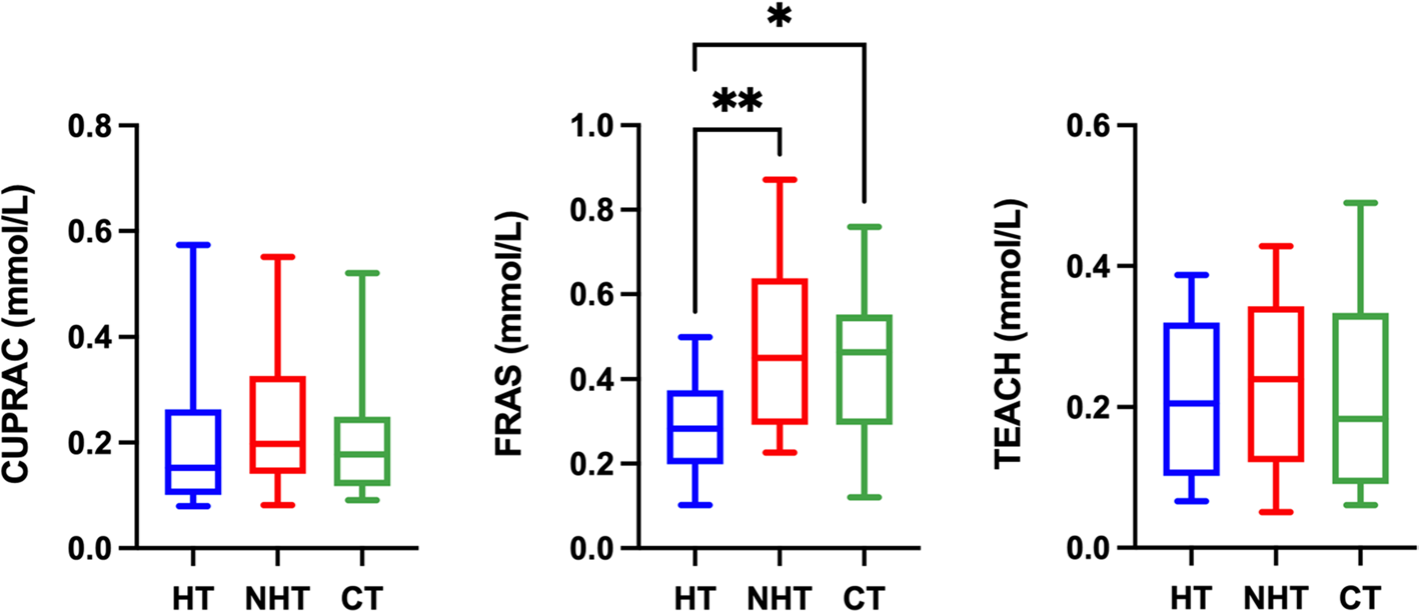
Results of the measurement of antioxidant biomarkers in saliva. Cupric reducing antioxidant capacity (CUPRAC); ferric reducing ability of saliva (FRAS), and Trolox equivalent antioxidant capacity (TEAC) in dogs with hypothyroidism (HT), non-thyroid diseased (NHT) dogs and controls (CT). Asterisks indicate significant differences between groups. *P﻿ ≤ 0.05, **P ﻿ ≤ 0.01

**Fig. 4:**
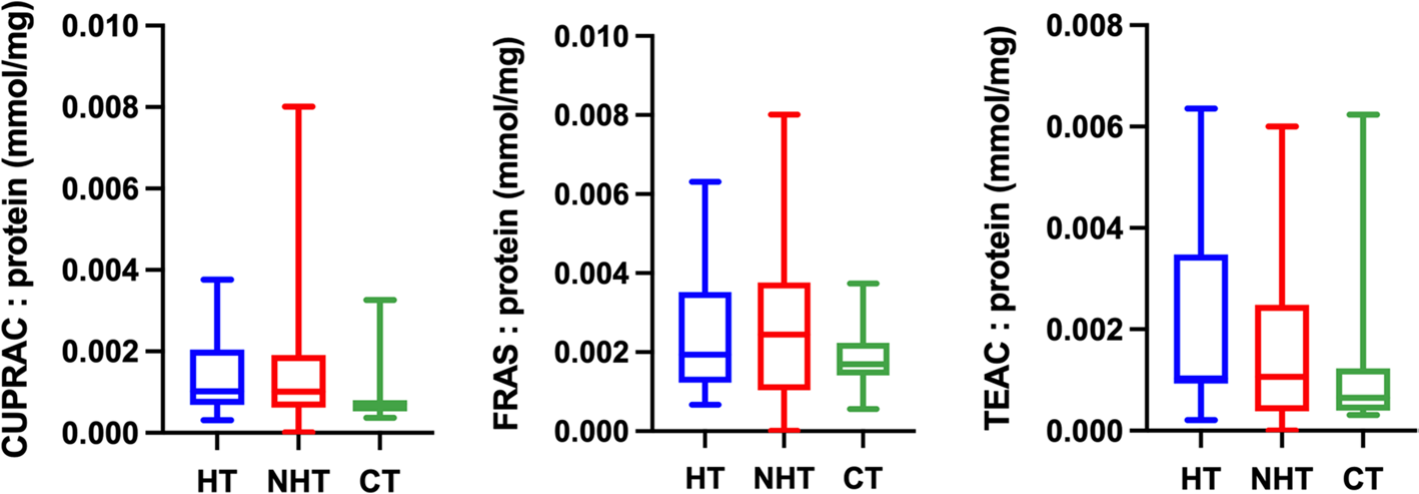
Ratio between the salivary antioxidants results and salivary protein concentration. Cupric reducing antioxidant capacity (CUPRAC), ferric reducing ability of saliva (FRAS), and Trolox equivalent antioxidant capacity (TEAC) in dogs with hypothyroidism (HT), non-thyroid diseased (NHT) dogs and controls (CT)

Results for the salivary antioxidant biomarkers are shown in Fig. 3. FRAS concentrations before protein correction were significantly lower in dogs with hypothyroidism in comparison to dogs with non-thyroid diseases (*P* = 0.0026) and healthy dogs (*P* = 0.0158). No statistical differences (*P* > 0.05) were found for CUPRAC and TEAC between groups. When the results were corrected by salivary protein, CUPRAC, FRAS, and TEAC showed no significant differences (*P* > 0.05) (Fig. 4).

#### Oxidant status

**Fig. 5:**
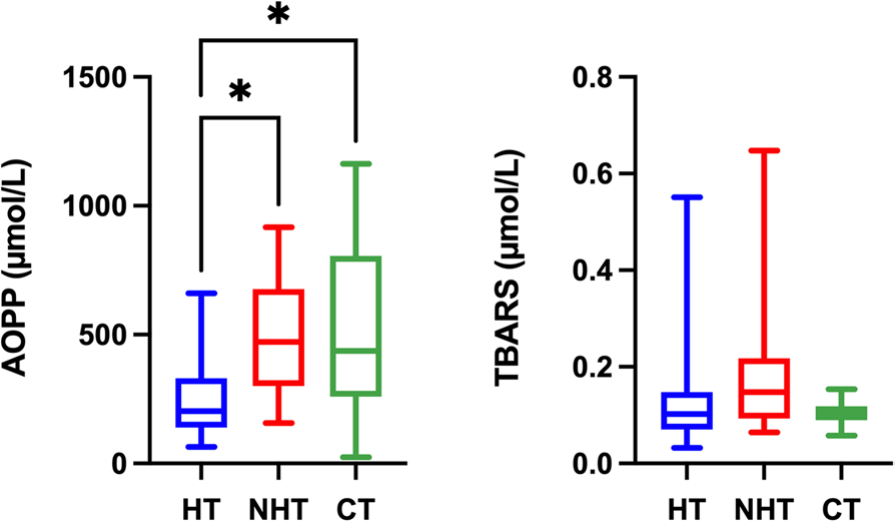
Results of oxidant biomarkers in saliva. Advanced oxidation protein products (AOPP), and thiobarbituric acid reactive substances (TBARS) in dogs with hypothyroidism (HT), non-thyroid diseased (NHT) dogs, and controls (CT). Asterisks indicate significant differences between groups. *P﻿ ≤ 0.05

**Fig. 6:**
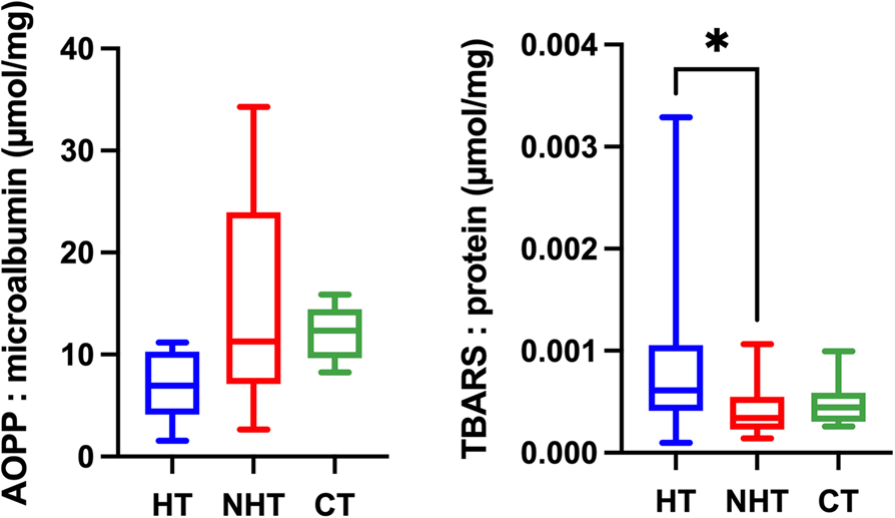
Ratio between the salivary antioxidants results and salivary protein or microalbumin concentration. Advanced oxidation protein products (AOPP), and thiobarbituric acid reactive substances (TBARS) in dogs with hypothyroidism (HT), non-thyroid diseased (NHT) dogs, and controls (CT). Asterisks indicate significant differences between groups. *P﻿ ≤ 0.05

Results for the salivary oxidant biomarkers are shown in Fig. 5 and 6. AOPP concentrations before microalbumin correction were significantly lower in dogs with hypothyroidism in comparison to dogs with non-thyroid diseases (*P* = 0.0113) and healthy dogs (*P* = 0.0113). When the AOPP results were corrected by salivary microalbumin no significant differences (*P* > 0.05) were found. No statistical differences (*P* > 0.05) were found for TBARS before protein correction; however, when the results were corrected by salivary protein concentration TBARS values were significantly higher in dogs with hypothyroidism in comparison to dogs with non-thyroid diseases (*P* = 0.0210).

### Correlation study

Correlations between results obtained in serum and saliva, and serum and corrected saliva are found in Table 2. Correlations between the results of serum AOPP and corrected serum AOPP based on its albumin concentrations, and salivary AOPP and corrected salivary AOPP based on its microalbumin concentrations are found in Table 3. As shown in Table 3, serum AOPP was correlated to serum  AOPP corrected by albumin (*r* = 0.4175, *P* = 0.0006), nonetheless, no significant correlation was found between any of the biomarkers measured in both samples.

**Table 2:** Correlation coefficients (*r*) between serum and saliva, and serum and corrected saliva based on its protein concentrations in dogs

Biomarker	Serum VS Saliva	Serum VS Corrected Saliva
CUPRAC	-0.0347	-0.1306
FRAP	-0.1248	-0.1358
TEAC	-0.2512	-0.2230
TBARS	-0.1782	-0.0275

*CUPRAC* Cupric reducing antioxidant capacity, *FRAP* Ferric reducing ability of plasma, *TEAC* Trolox equivalent antioxidant capacity, *TBARS* thiobarbituric acid reactive substances

**Table 3:** Correlation coefficients between serum and salivary AOPP concentrations, using obtained values and values corrected by albumin for serum (CSe) and microalbumin for saliva (CSa)

Comparison	Correlation coefficient (*r*)
Serum VS CSe	0.4175 ***
Saliva VS CSa	0.7336
CSe VS Saliva	0.1268
CSe VS CSa	0.9393

Asterisks indicate significant differences

^***^P﻿ ≤ 0.0002

## Discussion

To the author’s knowledge, there are no reports about biomarkers of the redox status in serum and saliva of dogs with hypothyroidism. In this study, changes in various antioxidant and oxidant biomarkers were found in hypothyroid dogs that could indicate an altered redox status in this disease.

An increase in three antioxidant biomarkers (TEAC, PON-1, and GPx) was found in serum of dogs with hypothyroidism. The increases in GPx are in line with what was described in humans with Hashimoto’s thyroiditis [16, 23–25]. GPx is an important selenoprotein that scavenges and detoxifies H_2_O_2_, protecting thyroid cells from oxidative damage [26–28]. We hypothesize that the increase in GPx activity could be associated by an increased TSH stimulation, as some reports indicate that this hormone can influence GPx activity by increasing its concentrations [29].

The increase in PON-1 in hypothyroid dogs of our study differs from the decreases described in humans with hypothyroidism; in which low PON-1 values are considered a risk of atherosclerosis associated with this disease [30–33]. Dogs carry cholesterol in HDL where PON-1 is bounded protecting it from oxidation [34] instead of in LDL, as it occurs in humans. Therefore, the elevation of cholesterol that usually appears in canine hypothyroidism could imply an increase in HDL and subsequently in PON-1. This could explain the increase of PON-1 found in dogs with hypothyroidism in our study, and this increase in PON-1 could also be one of the reasons why dogs have lower risks of atherosclerosis than humans [35–37]. In humans, TEAC was found to be decreased in various thyroid disorders, including multinodular goitre, contrary to our results [38]. Since TEAC is an analyte that represents the effect of various antioxidants, we believe further studies should be made to evaluate if PON-1 and/or GPx influence the concentrations of TEAC. In addition, alpha-tocopherol, one of the molecules measured by TEAC, is known to be transported through lipoproteins which are increased in hypothyroidism. Although it was not evaluated in this study, increases in this antioxidant are associated with increases in lipoproteins which could explain the high TEAC levels found in this study [39].

On the other hand, in serum, three oxidant biomarkers were found to be increased in dogs with hypothyroidism, namely, TOS, POX-Act, and d-ROMs. TOS measures the total oxidant status, as it is integrated by different oxidant molecules [40]. In humans, serum TOS concentrations were found to be increased in subclinical hypothyroidism [41] and Hashimoto’s thyroiditis [42]. The increases in POX-Act and d-ROMs could be related to the increase in TOS concentrations, as both oxidants are part of the whole oxidant status of an organism [40, 43, 44].

In our study, serum AOPP concentrations without albumin correction were decreased in hypothyroid patients, however, when values were corrected, no significant changes were found, as reported in humans [1, 45]. In general, it is recommended to correct serum AOPP concentrations by albumin concentrations, as AOPP are carried by oxidized plasma proteins like albumin [46, 47], so it could be postulated that the corrected AOPP values should be considered for studies.

In saliva, FRAS and AOPP were decreased in hypothyroid dogs. However, when the salivary biomarkers were corrected by protein, no significant changes were found in these analytes. On the other hand, the salivary TBARS: protein ratio increased in dogs with hypothyroidism. Although this is a topic that should be discussed more deeply in the future, it seems that the results of saliva after correction would be more in line with what has been described in humans, since TBARS concentrations have been shown to be increased in plasma [23] and erythrocytes [48], possibly due to an increase in lipid peroxidation that is associated with the hyperlipidaemia consistently seen in hypothyroidism [49, 50]. In our study, no significant changes were observed in TBARS serum or saliva without correction in dogs with hypothyroidism. Therefore, it could be suggested that for TBARS, the correction of salivary results by protein could be more sensitive to detect the oxidant overproduction occurring in dogs with hypothyroidism, which is evidenced by the increases in TOS, POX-Act, and d-ROMs in serum. However, further studies should be performed using a larger number of animals and ideally also in dogs going through levothyroxine replacement therapy to confirm the results of our report. The divergences observed between serum and salivary TBARS are in line with the lack of correlation shown, as well as in the other serum and saliva biomarkers of redox status of our study, as divergencies in the composition of both fluids have been described in other reports in dogs [51], pigs [52] and humans [53].

This report should be considered a preliminary study, and, as previously mentioned, further trials with larger populations should be made to evaluate the changes in the oxidative status analytes used in this study throughout treatment. Also, an evaluation of the biomarkers in other sample types, such as whole blood and red blood cell lysates should be performed, since in a previous study these samples provided additional interesting information about the redox status in dogs [54]. In addition, further studies should be made to evaluate the applicability of these biomarkers in the diagnosis of this disease and treatment monitorization. These studies should assess if the presence of changes found in serum and saliva in dogs with clinical signs compatible with hypothyroidism could possibly help on the suspicion of this disease and also evaluate if the normalization of these biomarkers could be a sign of an adequate treatment.

## Conclusions

Biomarkers of redox status show changes in serum and saliva in canine hypothyroidism compared with both healthy and non-thyroid-diseased dogs. Namely, there was an increase of antioxidants (TEAC, PON-1, and GPx) and oxidants (TOS, POX-Act, and d-ROMs) in serum of dogs and an increase in TBARS: protein ratio in dogs with hypothyroidism in comparison to healthy dogs. However, further studies should be made to confirm our results in larger populations of dogs and evaluate the practical potential of these analytes as biomarkers for this disease.

## Methods

### Study design

This case–control study involved client-owned dogs attending different Veterinary Clinics of Murcia Region (Spain) between March 2021 and May 2022.

Dogs were divided into three groups. A group of dogs with hypothyroidism (*n* = 23) following the subsequent criteria: (1) being adult dogs (over a year old), (2) not presenting other diseases, (3) not having received any treatment six months before diagnosis, (4) presence of symptoms related to the disease that justify the specific diagnosis tests (lethargy, tiredness, weight gain) [55] and (5) having the disease confirmed through specific diagnostic tests (determination of T4 and thyroid stimulating hormone (TSH)) [56]. Secondly, a group of non-thyroid diseased dogs (*n* = 21) with the same criteria mentioned previously was included, except for having negative results for specific thyroid diagnostic tests [56], and lastly, a control group of healthy adult dogs (*n* = 16) that were ruled out of any disease were included. In all cases, the nutritional status was reported based on the body condition score (BCS) 5-scale (1-thin; 2-underweight; 3-optimal (lean); 4-overweight; 5-obese) [57].

Samples were obtained after 12 h of fasting. Blood samples were obtained by jugular venipuncture after the saliva collection. In brief, saliva was obtained by introducing a sponge into the dog’s mouth for one to two minutes until wet and then placed into a Salivette tube (Salivette, Sarstedt, Aktiengesellschaft & CO., Nümbrecht, Germany). All tubes with blood and saliva were stored with ice until taken to the laboratory, where they were centrifuged at 3000 × *g* for 20 min at 4 ºC. Obtained serum and saliva were collected and transferred into 1.5 mL tubes and stored at -80ºC until analysis. All samples were obtained by the clinicians and were sent to our laboratory for further analysis. None of the dogs used in this study showed any clinical signs of periodontitis. Informed consent was taken from the dog owners.

### Assays

#### Antioxidant status

The determination of the CUPRAC assay was based on the reduction of Cu^2+^ into Cu^1+^ by the nonenzymatic antioxidants in the sample [58]. Evaluation of CUPRAC was made following the procedure previously validated for use in serum of dogs [59]. Results are expressed in millimoles per liter (mmol/L). CUPRAC was measured in serum and saliva.

Determination of the FRAP/FRAS assay was based on the reduction of ferric-tripyridyltriazine (Fe^3+^-TPTZ) to the ferrous (Fe^2+^) form [60] by the sample. Its determination was made following previously described methods [60, 61]. Results are expressed in mmol/L. This assay was measured in serum (FRAP) and saliva (FRAS).

Measurement of TEAC was based on the assay described by Arnao et al. [62] that has been used previously in canine serum samples [63]. Its principle is based on the enzymatic generation of 2,2’-azino-bis(3-ethylbenz-thiazoline-6-sulfonic acid) (ABTS) radical and its reduction by non-enzymatic antioxidants present in the sample [62]. Results are expressed in mmol/L. TEAC was measured in serum and saliva.

The determination of total thiol is based on the reaction of thiols within the sample with 5,5’-dithiobis-(2-nitrobenzoic acid) (DTNB). The assay used was performed according to previously described methods for serum samples [64, 65]. Results are expressed in micromoles per liter (µmol/L). Thiol was measured in serum but could not be detected in saliva.

Measurement of PON-1 was based on the hydrolysis of phenyl acetate into phenol and it was determined as previously described in canine serum [66]. Results are expressed in units per milliliter (IU/ml). PON-1 was measured in serum but could not be measured in saliva.

Measurement of GPx was based on the use of commercially available assays following the manufacturer’s instructions (Randox, Crumlin, UK), as used in previous studies [67, 68]. Results are expressed in IU/ml. GPx was measured in serum but could not be measured in saliva.

### Oxidant status

Determination of TOS was based on the assay described by Erel [40] that was previously used in dogs [69]. Its reaction is based on the ability of oxidants in the sample to oxidize Fe ^2+^-*o*-dianisidine complex to Fe^3+^ [40]. Results are expressed in µmol/L. TOS was measured in serum but could not be detected in saliva.

Evaluation of the POX-Act assay was based on the determination of total peroxides through a peroxide-peroxidase reaction using tetramethylbenzidine as the chromogenic substrate [44]. Determination of POX-Act was measured following a validated method for human sera [44]. Results are expressed in µmol/L. POX-Act was measured in serum but could not be measured in saliva.

Measurement of the d-ROMs assay was based on the reaction of the sample in an acidic medium in the presence of *N*,*N*,-diethyl-*para*-phenylenediamine (DEPPD), and it was made following a previously described method [43]. Results are expressed in Carratelli Units (U.CARR). d-ROMs was measured in serum but could not be measured in saliva.

Determination of AOPP was based on oxidized albumin and di-tyrosine containing cross-linked proteins, as previously described [47], and measured in canine serum [70]. Results are expressed in µmol/L. AOPP was measured in serum and saliva.

Determination of TBARS is based on the reaction of the sample to a Trichloroacetic acid, thiobarbituric acid, and *N* hydrochloric acid in heated conditions [71]. TBARS was measured following a previously described method [71] using a microplate reader (Powerwave XS, Biotek Instruments). Results are expressed in µmol/L. TBARS was measured in serum and saliva.

### Proteins

Salivary protein concentration was measured using a commercially available colorimetric kit (protein in urine and CSF, Spinreact, Spain) as done in previous studies [72]. Results are expressed in milligrams per milliliter (mg/mL).

Microalbumin was measured in saliva using a commercially available (Microalbumin spectrophotometry kit, REF 22,324, Biosystems, Barcelona, Spain). Results are expressed in milligrams per deciliter (mg/dL).

Salivary values for all assays were corrected according to total protein concentrations found on each saliva sample as performed previously [73], meanwhile, salivary AOPP values were corrected using salivary microalbumin concentrations, as well as serum AOPP values were corrected using serum albumin concentrations, according to previous reports [46, 47].

### Statistical analysis

Data were analyzed using GraphPad Prism software (GraphPad Software Inc., version 9.3 for MacOS). The Shapiro–Wilk test was first used to assess whether the results for each analyte were normally distributed. Differences in the concentrations between groups, when data were normally distributed, were assessed using a One-way ANOVA followed by Tukey’s multiple comparisons range test, and non-normally distributed data were assessed with a One-way ANOVA followed by the Kruskal–Wallis test. Correlation between saliva and serum was assessed using Pearson’s and Spearman’s correlation tests, according if the data were normally distributed or not. Statistical differences were considered for *P*-values < 0.05. Parametric data is shown as mean ± standard deviation (SD), meanwhile, non-parametric data is shown as median and interquartile range (IQR).

## Supplementary Information

**Additional file 1:** **Table S1.** Description of the methods and the reagents used on the assays performed in the study.

## Data Availability

The datasets generated and/or analyzed during the current study are available from the corresponding author upon reasonable request.

## Abbreviations

T4: Tetraiodothyronine
T3: Triiodothyronine
ROS: Reactive oxygen species
SOD: Superoxide dismutase activity
TBARS: Thiobarbituric acid reactive substances
CUPRAC: Cupric reducing antioxidant capacity
FRAP: Ferric reducing ability of plasma
FRAS: Ferric reducing ability of saliva
PON-1: Paraoxonase type-1
GPx: Glutathione peroxidase activity
TOS: Total oxidant status
POX-Act: Peroxide-activity
d-ROMs: Reactive oxygen-derived compounds
AOPP: Advanced oxidation protein products
TSH: Thyroid stimulating hormone;
BCS: Body condition score
ABTS: 2,2’-Azino-bis(3-ethylbenz-thiazoline-6-sulfonic acid)
DTNB: Dithiobis-(2-nitrobenzoic acid)
DEPPD: *N*,*N*,-Diethyl-*para*-phenylenediamine
