# Supplementary material for: Changes in biomarkers of redox status in serum and saliva of dogs with hypothyroidism
Source: BMC Vet Res. 2023 Feb 3;19:33. doi: 10.1186/s12917-023-03586-4 (PMC9896751; doi:10.1186/s12917-023-03586-4)
Supplement: Supplementary file 1 — Additional file 1: Table S1. Description of the methods and the reagents used on the assays performed in the study. [file 12917_2023_3586_MOESM1_ESM.docx]

**Table S1:** Description of the methods and the reagents used on the assays performed in the study.

| **Method** | **Basis of the method** | **Reagents (in home-made assays) or manufacture** | | |
| --- | --- | --- | --- | --- |
|  |  | **Reagent 1** | | **Reagent 2** |
| **CUPRAC** | The reduction of Cu2+ to Cu1+ caused by antioxidants in the sample | bathocuproinedisulfonic acid disodium salt in phosphate buffer | | Cu(II) sulphate anhydrous in ultrapure water |
| **FRAP/FRAS** | The reduction of Fe3+ to Fe2+ caused by antioxidants in the sample | tripyridyltriazine, and ferric chloride hexahydrate in acetate buffer | | none |
| **TEAC** | The reduction of 2,2′-azino- bis(3-ethylbenzthiazoline-6-sulfonic acid) (ABTS) radical to ABTS caused by the antioxidants present in the sample | ABTS, horseradish peroxidase (HRP), and H2O2 in phosphate buffer | | none |
| **Thiol** | Reaction of thiols in the sample with ﻿5,5’-dithiobis-(2-nitrobenzoic acid) (DTNB) | Tris – EDTA Buffer | | DTNB in phosphate buffer |
| **PON-1** | Hydrolysis of phenylacetate into phenol | Tris and CaCl_2_  buffer | | p-nytrophenyl acetate and methanol solution diluted with ultrapure water |
| **GPx** | Catalysis of the oxidation of glutathione by cumene hydroperoxide, and the reduction of the oxidise glutathione by presence of glutathione reductase | RANDOX | | |
| **TOS** | Estimation of the oxidation of ﻿Fe2+-o-dianisidine complexes to Fe3+ within the sample | Xylenol orange tetrasodium salt, NaCl and glycerol in H_2_SO_4_ solution | | Ferrous ammonium sulfate and o-dianisidine dihydrochloride in H_2_SO_4_ solution |
| **POX-Act** | Measurement of total peroxides after a peroxide-peroxdiase reactiong using a chromogenic substrate; tetramethylbenzidine (TMB) | TMB and HRP in acetate buffer | | none |
| **d-ROMs** | Based on Fenton’s reaction. Estimation of total peroxides by evaluating the N,N-dyethylparaphenyldiamine (DEPPD) radical cation concentration | Acetate buffer | | DEPPD in ultrapure water |
| **AOPP** | Absorbance of oxidatively-modified albumin at 340 nm in the presence of potassium iodide (KI) in acidic conditions | KI in ultrapure water | | Acetic acid |
| **TBARS** | Reaction of the sample to a trichloroacetic acid, thiobarbituric acid and ﻿N hydrochloric acid stock in heated conditions. | ﻿ trichloroacetic acid; thiobarbituric acid; hydrochloric acid | none | |

All assays except TBARS, were conducted in the Olympus AU400, at a constant temperature of 37°C and in a reaction time between 5 and 8 minutes. Evaluation of TBARS was made manually using a microplate reader (Powerwave, XS, Biotek instruments).
